# Can rhythm therapy cure valvular disease?

**DOI:** 10.1007/s10840-023-01487-y

**Published:** 2023-01-27

**Authors:** Stephan H. Schirmer, Robert Bernat

**Affiliations:** 1 Kardiopraxis Schirmer, Am Altenhof 8, 67655 Kaiserslautern, Germany; 2 Saarland University, Homburg/Saar, Germany; 3 Marienhospital Aachen, Aachen, Germany; 4 Unversität Osijek, Osijek, Croatia

Atrial fibrillation (AF) is the most common rhythm disorder worldwide, with increasing prevalence. It is associated with significant morbidity through thromboembolism and stroke, but also myocardial remodeling. Registry data indicate that almost 2/3 of the AF patients have significant valvular heart disease (VHD) [1]. There is a bidirectional connection between AF and VHD, in which the causal relationship cannot always be determined. AF is particularly associated with functional tricuspid regurgitation (TR), leaving the structure of the leaflets and chordae normal, whereas regurgitation is primarily caused by changes of the surrounding structures—annular dilatation and dysfunction of the right ventricle [2]. Higher grade TR, although often clinically silent for a long time, can cause severe problems of right heart failure, congestion, and also mortality. The relationship between AF and TR has been known for more than half a century [3], yet the concept of atrial function and tricuspid regurgitation (AF-TR), as opposed to the well-studied concept of TR based on RV enlargement and dysfunction, has largely been understudied and ignored.

Tricuspid valve disease is often under-recognized and underestimated because of the lack of treatment options, although recently, also the tricuspid valve is amenable to modern interventional techniques such as transcatheter edge-to-edge repair (TEER), following years of mitral valve experience. Therefore, elimination or at least reduction of one of the causes of functional TR, i.e., atrial fibrillation, might have an important effect on valve function and potentially also clinical endpoints. The current guidelines on AF and VHD do not address the interplay between the two conditions, making the growing body of data on influencing the course of TR by treating AF even more important. The effects of different treatment options for AF on TR have been primarily examined in retrospective studies. A recent investigation points towards one-third of patients with newly diagnosed AF developing moderate or greater TI which was associated with increased risk of mortality in a 13-year follow-up [4]. Some studies have indicated that successful catheter ablation of atrial fibrillation with durable sinus rhythm may lead to improvement of tricuspid valve function in case of lesser tethering height (< 6 mm) [5].

In this issue of the journal, the authors demonstrate marked improvement of formerly severe functional TI in a retrospective cohort analysis of *n* = 64 patients undergoing catheter ablation of AF [6]. They derived the carefully selected cohort from a large single-center database of > 2.000 patients undergoing ablation, of whom the majority did not suffer from severe TI. Moreover, the area of right ventricle and atrium as well as mitral regurgitant jet area improved alongside. Because of this large database and the clear results, the analysis, albeit retrospective in nature, is helpful for clinical evaluation, and the authors should be congratulated on performing the investigation. Patients with persistent AF and only mild symptoms are common, and the decision for an interventional approach of putative prognostic value is not always easy to make. The early treatment of AF fits well in the widely advocated strategy of timely TR treatment, regardless of the exact causal relationship between the two entities [7].

Yet, the study is somewhat confirmative, although past investigations have included less patients and a lesser degree of severity of TR [8]. In addition, other studies focused on the differences between surgical and catheter ablation and not so much on echocardiographic parameters of TR. A retrospective analysis on > 400 patients undergoing surgical AF ablation during mitral valve surgery confirmed AF recurrence to be associated with progression of TR and right remodeling TR [5]. Not only improvement of the degree of tricuspid regurgitation but also amelioration of right ventricular geometry is reported following catheter ablation of persistent AF. As with other reports, due to its retrospective and single center nature, the conclusions which can be deducted from the present investigation are limited. Ideally, a prospective study is necessary to reliably evaluate the effect of catheter ablation of AF on functional TR. Beyond this, we can only assume that the amelioration of echocardiographic parameters translates into clinical benefit—showing a reduction of hard clinical endpoints such as right heart decompensation, hospitalization, or even death would be the ultimate goal.

In summary, the current analysis adds to the body of (mostly retrospective) data suggesting a beneficial effect of rhythm therapy on functional right heart parameters, particularly tricuspid regurgitation. While there are clear general recommendations on the early and aggressive use of catheter ablation in treating AF, its adjunct positive effects on TR, as documented in the present paper, add to this concept.
